# A method for multi-simulator reaction-diffusion with NEURON

**DOI:** 10.1186/1471-2202-15-S1-P109

**Published:** 2014-07-21

**Authors:** Robert A McDougal, Michael L Hines, William W Lytton

**Affiliations:** 1 Neurobiology, Yale University, New Haven, CT 06520, USA; 2 Physiology & Pharmacology, SUNY Downstate Medical Center, Brooklyn, NY 11203, USA; 3 Kings County Hospital, Brooklyn, NY 11203, USA

## 

There are two major approaches in the development of simulation tools for multi-scale modeling -- the all-in-one simulator; and the unifying simulation environment that allows multiple plug-ins to be utilized in order to provide different aspects of a simulation. While NEURON has traditionally taken the former approach, it has become clear that there are always going to be multiple approaches even to modeling at a single level, making it desirable to be able to readily plugin in external simulators to provide improvements or to try comparisons. For example, at the level of reaction-diffusion, the built-in scheme provides techniques that differ somewhat from those of Virtual Cell [1] or XTW [2], both of which are now available as plug-in environments.

NEURON is a widely used simulator for biophysically detailed neuronal electrophysiology models that has recently been extended with syntax for reaction-diffusion dynamics [3]. This class of problems is useful for studying the intracellular dynamics of neurons – e.g. protein-protein interactions or calcium-induced calcium release (CICR) – and how they interact with the electrophysiology. As many molecules play a role in both intracellular dynamics and electrophysiology, these must be simulated simultaneously. The developmental branch of NEURON offers experimental 3D reaction-diffusion support, but its performance remains below that of dedicated reaction-diffusion simulators.

To leap-frog over these performance issues, we have developed a new method for multi-simulator studies. In our technique, all model specification and simulation protocols remain specified in NEURON. Thus, switching between NEURON’s experimental integrated 3D solver and an external solver requires only adding the connection code. No familiarity with the external solver is required in contrast to multi-simulations with MUSIC [4], where the models are described using each simulator’s native description. The simulators run in different processes. All model specification, currents, and concentration information is exchanged via shared memory. As an example, we demonstrate using our technique to study the behavior of CICR waves near the soma of a pyramidal neuron.
